# NAD ^+^ biosynthesis, aging, and disease

**DOI:** 10.12688/f1000research.12120.1

**Published:** 2018-02-01

**Authors:** Sean Johnson, Shin–ichiro Imai

**Affiliations:** 1 Department of Developmental Biology, Washington University School of Medicine, St. Louis, USA

**Keywords:** NAD+, Biosynthesis Aging

## Abstract

Nicotinamide adenine dinucleotide (NAD ^+^) biosynthesis and its regulation have recently been attracting markedly increasing interest. Aging is marked by a systemic decrease in NAD ^+^ across multiple tissues. The dysfunction of NAD ^+^ biosynthesis plays a critical role in the pathophysiologies of multiple diseases, including age-associated metabolic disorders, neurodegenerative diseases, and mental disorders. As downstream effectors, NAD ^+^-dependent enzymes, such as sirtuins, are involved in the progression of such disorders. These recent studies implicate NAD ^+^ biosynthesis as a potential target for preventing and treating age-associated diseases. Indeed, new studies have demonstrated the therapeutic potential of supplementing NAD ^+^ intermediates, such as nicotinamide mononucleotide and nicotinamide riboside, providing a proof of concept for the development of an effective anti-aging intervention.

## Introduction

In recent years, interest in nicotinamide adenine dinucleotide (NAD ^+^) biology has significantly increased in many different fields of biomedical research. A number of new studies have revealed the importance of NAD ^+^ biosynthesis for the pathophysiologies of aging and aging-related diseases. This short review will highlight the recent progress in this new connection between NAD ^+^ biosynthesis, aging, and disease. In particular, we will focus on the role of NAD ^+^ in aging and longevity control, its effect on the function of NAD ^+^-dependent enzymes such as sirtuins, and its relation to the development and progression of age-associated disorders. Finally, we will address the preventive and therapeutic potential of NAD ^+^ intermediates. 

## NAD ^+^ biosynthetic pathways

NAD ^+^ is an essential component of cellular processes necessary to support various metabolic functions ^1– 5^. The classic role of NAD ^+^ is a co-enzyme that catalyzes cellular redox reactions, becoming reduced to NADH, in many fundamental metabolic processes, such as glycolysis, fatty acid beta oxidation, or the tricarboxylic acid cycle ^6– 8^. In addition to playing these roles, NAD ^+^ has a critical role as the substrate of NAD ^+^-consuming enzymes such as poly-ADP-ribose polymerases (PARPs), sirtuins, and CD38/157 ectoenzymes ^9– 11^. These NAD ^+^-consuming enzymes have been known to mediate many fundamental cellular processes ^5^.

There are five major precursors and intermediates to synthesize NAD ^+^: tryptophan, nicotinamide, nicotinic acid, nicotinamide riboside (NR), and nicotinamide mononucleotide (NMN). NAD ^+^ can be synthesized *de novo* by the conversion of the amino acid tryptophan through multiple enzymatic steps to nicotinic acid mononucleotide (NaMN) ^12, 13^. NaMN is converted to nicotinic acid dinucleotide (NaAD ^+^) by NMN/NaMN adenylyltransferases (NMNATs) and then amidated to NAD ^+^ by NAD ^+^ synthetase.

**Figure 1.**
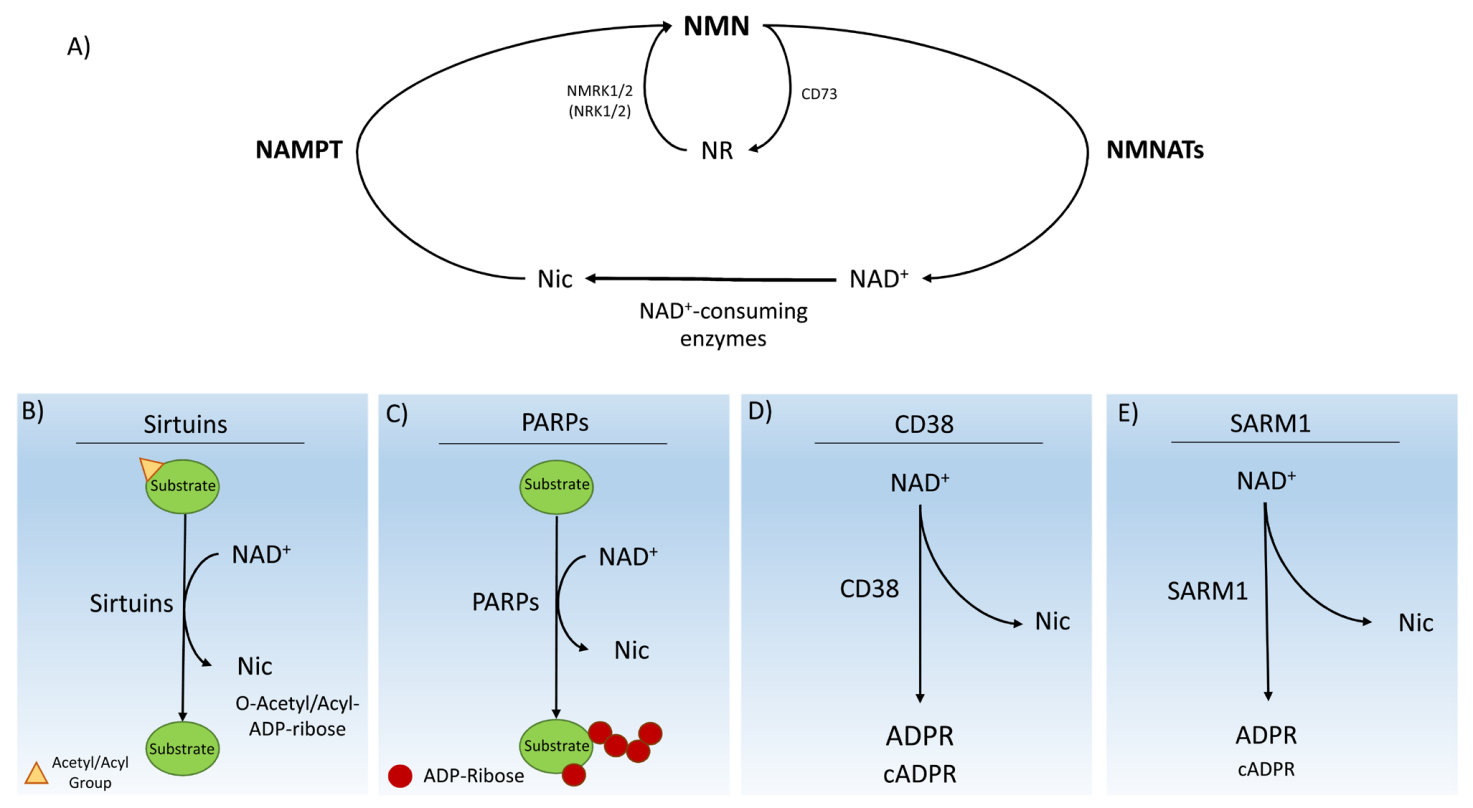
The major nicotinamide adenine dinucleotide (NAD ^+^) biosynthetic pathway and NAD ^+^-consuming enzymes in mammals. ( **A**) The NAD ^+^ biosynthetic pathway from the precursor nicotinamide (NIC). The pathway is mediated by nicotinamide phosphoribosyltransferase (NAMPT), which produces nicotinamide mononucleotide (NMN). NMN is immediately converted to NAD ^+^ by NMN adenylyltransferases (NMNATs). Multiple enzymes consume NAD ^+^, producing nicotinamide and various products. NIC can be salvaged to begin the biosynthetic pathway again. NMRK1 and NMRK2 (also known as NRK1 and NRK2), as well as CD73, can produce NMN and NR. **(B)** Enzymatic activity of sirtuins. The most common enzymatic reaction performed by sirtuins is the deacetylation of acetylated substrate proteins. The resulting products from the consumption of NAD ^+^ are NIC and O-acetyl-ADP-ribose. Sirtuins can also catalyze several other deacylation reactions. ( **C**) Enzymatic activity of poly-ADP-ribose polymerases (PARPs). In response to DNA damage, PARPs synthesize poly-ADP-ribose chains on a variety of target proteins, including itself, to act as a signal for DNA repair enzymes. The reaction produces the ADP-ribose chains and NIC. ( **D**) Enzymatic activity of CD38. The CD38 ectoenzyme catalyzes the synthesis of ADP-ribose (ADPR) or cyclic ADPR (cADPR) from NAD ^+^. ( **E**) Enzymatic activity of SARM1. A newly discovered class of NADase, SARM1, consumes axonal NAD ^+^ after injury, catalyzing the synthesis of ADPR and NIC as well as a small amount of cADPR.

In mammals, a major pathway of NAD ^+^ biosynthesis is the salvage pathway from nicotinamide ( Figure 1). Nicotinamide is converted to NMN, a key NAD ^+^ intermediate, by nicotinamide phosphoribosyltransferase (NAMPT), the rate-limiting enzyme in this pathway ^12^. NMNATs then convert NMN into NAD ^+^^14, 15^. NAMPT plays a critical role in regulating cellular NAD ^+^ levels ^12, 13^. On the other hand, nicotinic acid is converted to NaMN by nicotinic acid phosphoribosyltransferase (NPT) ^12, 14, 15^. NR needs to be converted to NMN by nicotinamide ribose kinases, NMRK1 and NMRK2 (also known as NRK1 and NRK2), which phosphorylate NR ^16^.

Maintenance of adequate NAD ^+^ biosynthesis is paramount for cell survival and function. Derailment from normal NAD ^+^ homeostasis substantially affects not only the NAD ^+^/NADH pool required for redox reactions but also activities of NAD ^+^-dependent enzymes for crucial cellular functions.

## Mediators of aging: NAD ^+^-dependent enzymes

It is now becoming a consensus that NAD ^+^ levels decline at cellular, tissue/organ, and organismal levels during the course of aging ^17^. Activities of NAD ^+^-consuming enzymes are affected by this NAD ^+^ decline, contributing to a broad range of age-associated pathophysiologies ^5, 18^.

Sirtuins are a family of NAD ^+^-dependent deacetylases/deacylases which have central roles in translating NAD ^+^ changes to the regulation of many regulatory proteins for metabolism, DNA repair, stress response, chromatin remodeling, circadian rhythm, and other cellular processes. Through the mediation of such broad functions, sirtuins are evolutionarily conserved regulators for aging and longevity in diverse organisms ^5, 18^. Mammals have seven sirtuin family members, SIRT1–7, among which SIRT1 is the ortholog of silent information regulator 2 (Sir2) in budding yeast ^11^. The various sirtuin family members have a number of enzymatic functions and are localized to different subcellular compartments ^19^. Briefly, SIRT1 is localized mainly to the nucleus but is also present in the cytosol ^20^. SIRT2 is present mainly in the cytosol but can also be present in the nucleus ^21^. SIRT3–5 are localized in the mitochondrial compartment ^22^. SIRT6 is localized in the nucleus as well, and SIRT7 is localized in the nucleolus ^23, 24^. Sirtuins are classified as class III histone deacetylases dependent on NAD ^+^. However, they target numerous non-histone proteins to alter their functions. Furthermore, sirtuins have other enzymatic activities, including demethylglutarylase and other lysine deacylase activities of SIRT4 ^25^, demalonylase and desuccinylase activities of SIRT5 ^26^, de-long chain fatty deacylase activity of SIRT6 ^27^, and ADP-ribosyltransferase activity of SIRT4/SIRT6 ^28, 29^. These various NAD ^+^-dependent functions of sirtuins place them at a key position for the regulation of aging and longevity in diverse organisms ^5, 18^. For example, we have demonstrated that brain-specific SIRT1-overexpressing (BRASTO) transgenic mice are able to delay the process of aging and extend life span ^30^. Whole-body SIRT6-overexpressing male mice also show life span extension ^31^.

PARPs also consume NAD ^+^, cleaving it into nicotinamide and ADP-ribose (ADPR) and producing a chain of ADPR. Among many PARP family members, PARP1 and 2 are major NAD ^+^ consumers in the nucleus, responding to DNA strand breaks and facilitating the DNA repair process ^32^. As NAD ^+^ is a common substrate between PARPs and SIRT1, there is a competition between their activities. PARP1/2 deletion is able to enhance the activity of SIRT1, resulting in the increases in mitochondrial content, fatty acid oxidation, and protection from diet-induced obesity ^33^. Whereas PARP1 deletion increases NAD ^+^ levels, PARP2 deletion increases *Sirt1* expression through its function to bind to the promoter of the *Sirt1* gene and repress its expression ^33^. During the course of aging, PARP activation, possibly due to constant DNA damage, appears to contribute to significant decreases in intracellular NAD ^+^, exacerbating the decrease in SIRT1 activity ^34^.

CD38, one of the primary NADases in mammals, can modulate the NAD ^+^ levels as observed in CD38-deficient mice ^35, 36^. Although the activity of CD38 mainly generates ADPR and nicotinamide by hydrolysis of NAD ^+^, it has a secondary role to mediate cellular signaling through the generation of cyclic ADPR (cADPR), a potent Ca ^2+^ inducer ^10^. The NADase activity of CD38 has been studied in depth ^35– 37^. CD38 can also degrade the NAD ^+^ precursors, NMN and NR, as well as NAD ^+^, thus modulating cellular NAD ^+^ content ^38, 39^. It has been reported that CD38 protein levels increase in multiple tissues and organs over age, contributing to NAD ^+^ decline ^40^. Therefore, CD38-dependent modulation of NAD ^+^ can alter the activity of SIRT1 and other sirtuins, as well as other NAD ^+^-consuming enzymes, and affect cellular signaling and metabolism ^36, 37^. Inhibiting CD38 can also promote NAD ^+^ levels and improve glucose and lipid metabolism ^41^.

A newly discovered class of NAD ^+^ hydrolases is sterile alpha and Toll/interleukin-1 receptor motif-containing 1 (SARM1) ^42^. SARM1 is central to the degeneration of axons after injury. Axonal injury is accompanied by a depletion of NAD ^+^, and loss of SARM1 function delays axonal degeneration. It has been shown that the Toll/interleukin-1 receptor (TIR) domain of SARM1 is responsible for the NAD ^+^ hydrolase activity and promotes axonal degeneration ^42^. This discovery opens a new opportunity to develop the treatment of axonopathy, brain injury, and other neurodegenerative diseases.

## NAD ^+^ decline as an important trigger for age-associated pathophysiologies

The decline in NAD ^+^ over age was originally recognized in mice overexpressing SIRT1 in pancreatic β cells (BESTO mice) ^43^. Young BESTO mice showed a significant improvement of glucose-stimulated insulin secretion. However, as they aged, this phenotype was completely lost. Interestingly, NMN supplementation was able to restore this phenotype in the aged BESTO mice and even improve glucose-stimulated insulin secretion in aged wild-type mice ^44^. Thus, NAD ^+ ^decline over age was the cause for the loss of the BESTO phenotype. These findings suggest that the reduction of the NAD ^+^ pool with age is responsible for the age-associated impairment of glucose-stimulated insulin secretion. Since this report, a number of studies have also found that NAD ^+^ declines over age in worms, flies, and mice ^5, 8, 17, 18^. Particularly in mice, it has been shown that several different tissues and organs show decreases in NAD ^+^ levels over age, causing metabolic dysfunctions, cardiovascular diseases, neurodegenerative disorders, and cancer ^17, 43– 45^.

A significant cause for this age-associated NAD ^+^ decline is the decrease in NAMPT-mediated NAD ^+^ biosynthesis. It has been shown that the expression of *Nampt* at both mRNA and protein levels is reduced over age in a variety of tissues ^45, 46^. This age-associated decrease in *Nampt* expression causes a reduction in NAD ^+^ in those same tissues, affecting the activities of NAD ^+^-dependent enzymes and redox reactions within the cell and leading to functional decline. Therefore, supplementation with NAD ^+^ intermediates, such as NMN and NR, can effectively restore the NAD ^+^ pool and cellular functions in aged animals.

Another cause for NAD ^+^ decline with age is the increase in NAD ^+^ consumption, and this is mainly due to the activation of PARPs ^33^. It has been reported that PARP1 activity increases, potentially due to the accumulation of DNA damage, so that more poly-ADP-ribose molecules are synthesized in aged tissues ^33^. This continuous PARP activation further depletes the NAD ^+^ pool and causes a reduction in the activity of SIRT1. Furthermore, ectopic PARP1 expression can cause multiple age-associated phenotypes ^47^. When PARP1 is knocked out, NAD ^+^ levels and SIRT1 activity significantly increase. Similar effects can be obtained by pharmacologically inhibiting PARP activity ^33^. The inhibition of PARP activity thus improves metabolic phenotypes through the activation of SIRT1. In contrast, it was recently reported that DNA damage repair decreases with age, along with a decrease in PARP1 activity ^48^. Interestingly, deleted in breast cancer 1 (DBC1) can bind to NAD ^+^ through its Nudix homology domain (NHD), which prevents it from binding to PARP1. As NAD ^+^ declines over age, DBC1 begins to bind to PARP1, reducing its DNA damage repair capacity ^49^. Therefore, it has been proposed that age-associated NAD ^+^ decline triggers the interaction between DBC1 and PARP1, contributing to the accumulation of DNA damage over age ^49^. Whether PARP1 is activated or inhibited over age could be cell type- or tissue-dependent, and further investigation will be required to clarify this contradiction. As mentioned above, the expression and activity of CD38 have been reported to increase with age ^40^. Indeed, CD38-deficient mice maintain NAD ^+ ^levels, mitochondrial respiration, and metabolic functions with age ^36^. Therefore, CD38 might have a significant contribution to age-associated NAD ^+^ decline in certain tissues.

The combination of decreased NAD ^+^ biosynthesis and increased NAD ^+^ consumption exacerbates the depletion of NAD ^+^, causing a variety of age-associated pathophysiologies ^43– 45^. Which one contributes further to the depletion of NAD ^+^ may be dependent on cell types and tissues. No matter what causes NAD ^+^ decline, it seems that major downstream mediators are sirtuins. The roles of sirtuins in the pathogenesis of age-associated diseases are summarized below.

## Diabetes

SIRT1 is important for promoting glucose-stimulated insulin secretion in pancreatic β-cells ^50, 51^. Additionally, SIRT1 has a protective effect against insulin resistance in peripheral tissues, including adipose tissue, liver, and skeletal muscle ^52^. These findings suggest that SIRT1 is important for glucose homeostasis and the prevention of type 2 diabetes. Whole-body *Sirt1*-overexpressing transgenic mice, when fed a high-fat diet (HFD), have shown improvements in glucose tolerance through reduction of hepatic glucose production ^52^. Additionally, these mice do not show changes in body weight or composition. In the kidney of diabetic model mice, SIRT1 inhibits oxidative stress, which can lead to nephropathy, by induction of cyclooxygenase-2 (COX-2) expression ^53^. It has also been shown that administration of NMN ameliorates glucose intolerance in HFD-induced type 2 diabetic mice, enhances hepatic insulin sensitivity, and restores oxidative stress gene expression, and inflammatory responses, partly through the activation of SIRT1 ^45^.

## Non-alcoholic fatty liver disease

Non-alcoholic fatty liver disease (NAFLD) is characterized by steatosis of the liver and is linked with insulin resistance and metabolic syndrome. Studies have observed a reduction of sirtuins in NAFLD ^54^. SIRT1/3/5/6 are reported to be reduced in patients with NAFLD ^54^. This reduction is accompanied by an increase in lipogenic genes such as fatty acid synthase and SREBP-1. SIRT1 and SIRT3 have particularly been investigated in regard to NAFLD. SIRT1 expression is reduced by HFD ^55^. Overexpression of SIRT1 upregulates fatty acid oxidation pathways and downregulates lipogenic pathways, protecting the liver from steatosis. SIRT3 function is impaired in HFD, leading to hyperacetylation of target proteins in the mitochondria and impairing their activities ^56– 58^. SIRT3-deficient mice exacerbate these phenotypes, while overexpression can ameliorate NAFLD ^59^.

## Atherosclerosis

SIRT1 has been shown to improve vascular function. SIRT1 is positioned to affect many pathways important for endothelial function ^60– 63^. SIRT1 suppresses the expression of inflammatory factors, including interleukin-6 (IL-6), monocyte chemoattractant protein 1 (MCP-1), intercellular adhesion molecule 1 (ICAM-1), matrix metalloproteinase 14 (MMP14), and vascular cell adhesion molecule 1 (VCAM-1) ^64^. Additionally, SIRT1 improves free fatty acid, triglyceride, total cholesterol, and blood glucose levels ^65, 66^. These protective effects of SIRT1 indicate that it acts as an anti-atherosclerosis agent. Consistent with these findings, NMN administration dramatically improves vascular function in aged mice, partly through the activation of SIRT1 ^67^.

## Alzheimer’s disease

Alzheimer’s disease (AD) is marked by multiple pathologies, including neuroinflammation, amyloid-beta plaques, mitochondrial damage, and increased oxidative stress ^68, 69^. Patients with AD have lowered expression of SIRT1 ^70, 71^, which is recapitulated in the hippocampus of AD model mice ^72– 75^. SIRT1 activation is capable of reducing the amount of oligomerized amyloid beta through upregulating the production of alpha-secretase ^68, 69^. This is corroborated by mouse models overexpressing SIRT1 and amyloid precursor protein. Additionally, SIRT1 promotes neuronal function and survival in AD model mice. CA1-localized SIRT1 overexpression not only preserves learning and memory in AD mice but enhances cognitive function in non-AD model mice ^76^.

## Retinal degeneration

Retinal degeneration is prominent in diseases such as macular degeneration and diabetic retinopathy. A recent study reported the importance of SIRT3 and SIRT5 in the survival of retinal photoreceptors ^77^. In particular, mitochondrial SIRT3 activity is sensitive to the reduction in NAD ^+^. Decreases in retinal NAD ^+^ were detected in multiple retinal degenerative disorders, including age-associated dysfunction, diabetic retinopathy, and light-induced degeneration ^77^. Supplementation with the NAD ^+^ intermediate NMN was able to restore retinal function ^77^. These findings suggest a possible therapeutic treatment for a wide variety of diseases with photoreceptor degeneration.

## Depression

Depression is a complex psychiatric disorder associated with a number of pathologies, including inflammation, synaptic dysfunction, metabolic syndrome, and cognitive deficit. Sirtuins have been shown to have a role in the development of depression ^78^. In the dentate gyrus region of the hippocampus, it has been shown that SIRT1 is decreased under conditions of chronic stress, which has been associated with depressive-like behaviors ^79^. Additionally, inhibition of SIRT1 by genetic or pharmacological methods has reproduced depressive behaviors. Activation of SIRT1 is able to lead to anti-depressive behaviors ^79^. However, it has been observed that SIRT1 regulates expression of monoamine oxidase A (MAO-A), which lowers serotonin and drives anxiety-like behaviors ^80^, indicating that a balance in SIRT1 expression/activity is important for mood disorders.

SIRT2 has also been reported in mood disorders. Hippocampal SIRT2 expression is decreased in chronic stress conditions ^81^. Pharmacological inhibition of SIRT2 recapitulates depressive behaviors. Adenovirus-mediated overexpression of SIRT2 produces anti-depressive behaviors, which were abolished when hippocampal neurogenesis was disrupted by X-irradiation ^81^.

## Interventions to achieve “productive aging”

NAD ^+^ intermediates, NMN and NR, are promising candidates to restore NAD ^+^ levels in disease models and aged animals ^17^. A number of studies have shown that both NAD ^+^ intermediates are effective to prevent and treat age-associated pathophysiologies.

We have shown that supplementation of NMN, a key NAD ^+^ intermediate, is effective at ameliorating age-associated metabolic disorders and slowing the progression of a multitude of age-associated physiological phenotypes ^45, 82^. Briefly, in the 12-month NMN administration study, age-associated body weight gain was ameliorated, energy metabolism and physical activity were improved, and gene expression changes associated with age were reversed. This study demonstrates NMN as an effective anti-aging agent ^82^. Other recent studies have also reported that NMN administration restores a depleted NAD ^+^ pool and is able to improve multiple aspects of disease. In a mouse AD model, one study reported that NMN improved mitochondrial respiration, a hallmark in the progression of AD and other neurodegenerative disorders ^83^. NMN administration has also shown improvements of mouse cognitive behaviors in the context of AD as well as improving electrophysiological deficits detected on hippocampal slices ^84, 85^. These findings suggest that NMN could also be a promising therapeutic agent for the treatment of AD and other neurodegenerative disorders. Additionally, we have shown the importance of NAD ^+^ biosynthesis in neuronal function. NAMPT is critical for neural stem cell proliferation and self-renewal. With age, NAMPT and NAD ^+^ levels decrease in the hippocampus, along with a decrease in the neural stem cell pool ^46^. NMN administration is able to rescue the NAD ^+^ levels and enhance the neural stem cell pool ^46^.

NR, another NAD ^+^ intermediate, has also shown beneficial effects in age-associated disorders. In prediabetic and diabetic mice under an HFD, NR administration improves steatosis of the liver, glucose tolerance, and weight gain ^86, 87^. These findings also suggest that NR administration could be an effective therapeutic agent for age-associated metabolic disorders. With age, the regenerative capacity of muscle decreases as muscle stem cells enter senescence. This is concomitant with a decrease in NAD ^+^ and a reduction of the mitochondrial unfolded protein response (mtUPR) ^88^. When NR is given, the muscle stem cell self-renewal capacity is restored, and the mtUPR is activated, improving the mitochondrial stress response. Additionally, in this study, mice which started receiving NR supplementation at two years of age showed a significant, moderate extension of life span ^88^. Dietary supplementation of NR significantly improves NAD ^+^ levels in the cerebral cortex and ameliorates cognitive deterioration ^89^. Application of NR in the context of hippocampal slice electrophysiology ameliorates deficits in long-term potentiation in the CA1 region. In this model system, NR increases PGC-1α, which regulates β-secretase and decreases amyloid-beta peptide. Though not addressed, the role of NAD ^+^-consuming enzymes could be central to these beneficial effects observed. It seems likely that NAD ^+^ depletion occurs in certain neurodegenerative diseases. Nuclear DNA damage has been suggested to be associated with neurodegenerative disorders ^90^. Thus, supplementation of NAD ^+^ intermediates, NMN and NR, would be effective agents to prevent and treat neurodegenerative disorders ( Table 1), and this is critical to achieve “productive aging”.

**Table 1.** Beneficial effects of supplementation of NAD ^+^ intermediates, such as nicotinamide mononucleotide and nicotinamide ribose.

	Phenotype	Normal progression	NAD ^+^ precursor intervention	Reference
Aging	Body weight	↑	↓	82
	Energy metabolism	↓	↑	82
	Mitochondrial function	↓	↑	82
	Insulin sensitivity	↓	↑	17, 82
Diabetes	Insulin sensitivity	↓	↑	17
	Glucose tolerance	↓	↑	17, 86, 87
	Oxidative stress response	↓	↑	86, 87
	Liver steatosis	↑	↓	86, 87
	Weight gain	↑	↓	86, 87
Alzheimer’s disease	Mitochondrial respiration	↓	↑	83
	PGC1α	↓	↑	89
	Beta-secretase	↑	↓	89
	Cognitive behaviors	↓	↑	84, 85, 89
	Long-term potentiation	↓	↑	84, 85

A more detailed summary is available in 17. NAD ^+^, nicotinamide adenine dinucleotide.

## Conclusions

It is now clear that systemic NAD ^+^ decline is one of the fundamental molecular events that regulate the process of aging and possibly limit organismal life span. NAD ^+^ biosynthesis particularly mediated by NAMPT and NAD ^+^ consumption by NAD ^+^-consuming enzymes are in a delicate balance so that perturbations to either side can cause significant derailment of the system. If NAMPT-mediated NAD ^+^ biosynthesis is disturbed or if NAD ^+ ^consumption is increased because of chronic DNA damage that elicits PARP activation, the intracellular NAD ^+^ pool is decreased, causing organismal functional decline. Different NAD ^+^-consuming enzymes, such as sirtuins, PARPs, CD38, and SARM1, might be affected in a cell type- or tissue-dependent manner, and loss of NAD ^+^ homeostasis can lead to dysfunction of basic physiological systems throughout the body. We now have increasing bodies of evidence supporting that interventions using NAD ^+^ intermediates, such as NMN and NR, can bolster the system by restoring the available NAD ^+^ and mitigate physiological decline associated with aging. We are at an exciting point in time when we can effectively test the importance of NAD ^+^ for the prevention and treatment of aging and aging-related diseases in humans.
